# Soil to plant transfer of alpha activity in potato plants: impact of phosphate fertilizers

**DOI:** 10.1186/s40201-015-0200-4

**Published:** 2015-05-16

**Authors:** Rishi Pal Chauhan, Amit Kumar

**Affiliations:** Department of Physics, National Institute of Technology, Kurkshetra, 136119 India

**Keywords:** Potato, Alpha radioactivity, LR-115, Phosphate fertilizers

## Abstract

**Background:**

Radionuclides in the phosphate fertilizers belonging to ^232^Th and ^238^U and ^40^ K are the major contributors to the outdoor terrestrial natural radiation. These radionuclides are transferred from fertilizer to food through soil.

**Materials and methods:**

Present work deals with the alpha activity in the different parts of the potato (*Solanum Tuberosum*) plants grown under controlled pots experiment using different amounts of phosphate fertilizers and urea. Alpha activities have been measured by track etch technique using the solid-state nuclear track detectors (LR-115).

**Results:**

Translocation factor for the fruit (edible Part) varied from 0.13 (for DAP) to 0.73 (for PF) with an average of 0.40 ± 0.26 for the plant grown with 20 g of fertilizers. Translocation factors increased with the increase in amount of fertilizers having value 0.51 ± 0.31 for the plant grown with 50 g of fertilizers. The translocation factor for the lower and the upper part of leaves varied from 0.44 to 0.67 and 0.22 to 0.83 with an average value 0.55 ± 0.15 and 0.45 ± 0.23 respectively. The transfer factor (*TF’s*) for the potato plants varied from 1.5 × 10^−2^ to 1.03 × 10^−1^ for root, from 1.3 × 10^−2^ to 1.23 × 10^−1^ for stem, from 2.1 × 10^−3^ to 4.5 × 10^−2^ for fruit and from 5.4 × 10^−3^ to 5.8 × 10^−3^ for lower part of the leaves after 105 days of the plantation.

**Conclusions:**

The results revealed that the alpha activity in the potato plants was higher in case of the plants grown with the use of phosphate fertilizers than with other fertilizers.

## Background

The accumulation of the radioactive elements in the environmental air and soil is of concern in relation to health of general public. The causes of radioactive contamination include accidental spills and emissions from nuclear-fuel operations, fallout from nuclear testing, and accidents at nuclear reactors. Radionuclides scattered into the environment are transported by air and water, ultimately reaching the soil and sediment, where they become bound [1]. The radioactive elements present in biosphere will involve with human through his food chain. Transportation of these radionuclides through soil to plant and then to food leads to uptake of radioactive elements to human. In biosphere these radioactive elements and their decay products get into the plants both through the above ground parts like leaves, and stem by external contamination and through the root spring up into the soil containing radioactive elements. The external contamination on aboveground parts is of less concerned as these are used in food after washing with water. The radioactive elements transfer from soil to plants root and then trans-located in different parts of plants depending upon the metabolism and condition of growth of plants [2]. ^238^U, ^226^Ra and ^232^Th concentrations in the soil and phosphate fertilizers are of vital importance, because through several pathways these radionuclides and their decays products will reach to human and increase the ingestion radiation doses. The pathways through which these radionuclides enter in biosphere are the use of fertilizers having high radioactivity contents [3]. The radionuclide contents of fertilizers varied directly with phosphate (P_2_O_5_) content [4].

### Fertilizers Impact in agriculture

In order to increase crop production and improving the properties of the nutrient-deficient lands, various chemical and phosphate fertilizers are now used. Miranzadeh et al. [5] reported that interactive application nitrogen fertilizer could have beneficial effects on wheat grain yield under similar agro-climatic conditions. The negative effect of the phosphate fertilizers on the agriculture use is the contamination of cultivated lands by trace metals (Cd, Cu and Zn) and increase in radioactivity in the vegetations and food [6–8]. Phosphate rock may be sedimentary, volcanic or biological origin is the starting material for all phosphate products including phosphate fertilizers [9]. The uranium, thorium and potassium contents of phosphate fertilizer depend upon the origin of phosphate rock varied from 10 to 5022 Bq/kg, 10 to 394 Bq/kg and 8 to 397 Bq/Kg respectively [10]. Phosphate rocks contain high concentration of ^238^U, ^226^Ra and ^232^ and their decays products due to accumulation of dissolved uranium during its formation [3]. The phosphate ore are used to produce phosphoric acid in Wet process by attack of the sulfuric acid (H_2_SO_4_). The ^238^U remains concentrated into phosphoric acid while ^226^Ra, ^210^Po, ^232^Th and ^210^Pb precipitated out as sulfate salt concentrated in phosphogypsum as the byproduct [11]. The uranium either in form of [U(SO_4_)_2_] or [UO_2_(SO_4_)] in phosphoric acid are water soluble, remain in phosphoric acid which is used for the fertilizers production, thus the uranium content of fertilizers expected to be high Khater [9]. Da Conceicao and Bonotto [12] reported the high concentration of ^232^Th and ^226^Ra in phosphoric acid than phosphogypsum.

There exist a linear relation between the P_2_O_5_ content and uranium in phosphate fertilizers [13]. The phosphorus is one of the 17 nutrients essential for plants growth. It involves in many chemical reactions occurring in plants for their growth like plant energy transfer, photosynthesis, genetic transfer and nutrient transports. It enters the plants through roots hair, root tips and outermost layers of root cells in the form of primary orthophosphate ion (H_2_PO_4_^−^) or secondary Orthophosphate (H_2_PO_4_^2−^). The stored phosphorus in plants root transports to other parts of plants, causing the mobility of uranium called translocation. The consumption of various parts of plants in food by man leads to ingestion of radiation dose. According to UNSCEAR [14] the uranium contents in the food varies from 1.2 Bq/kg to 400 Bq/kg for grain products and 9.8 Bq/kg to 400 Bq/kg for leafy vegetables. Plants metabolism plays important role in regulating the trace radioactive elements in the soil-plant-animal food chain system. Once non-nutrient radioactive trace elements are solubilized in water, plants root actively accumulate them depending upon chemical activity of the element in soil solution, the presence of competing ions, the redox potential and absorption capacity of the root. After absorption in the plant, trace elements are translocated, metabolized and stored in different part of plants depending upon the properties of the element as well as plant [15]. The transfer of elements from soil to plants assumed to be constant at low content in soil and vary for high content [16].

### Need of the study

The presence of uranium, radium, thorium and potassium in the plants enhance the gamma and alpha dose to the users: animal and man. The gamma spectroscopy or alpha spectroscopy applied for the measurements of these radio-activity in soil and different parts of plant enhanced by the use of phosphate fertilizers. The gamma spectroscopy uses the High Purity Germanium detector for determination of contents of different radionuclides like radium, polonium and lead ([17]. The alpha emitting nature of radium, polonium and bismuth make it possible to use alpha spectrometry for the determination of radioactivity contents used by Rodrıguez et al. [18]. The collective dose from the plants may be calculated by the total dose received by all the radionuclides present in the various parts of plants. The radionuclides present in the plants cause an alpha activity, which is measured by the track etch technique using solid state nuclear tracks detector (LR-115) used in present study.

### Assessment parameters

The assessment of the impact of these radionuclides on human can be derived from the estimation of plant uptake from the soil and applying food-chain model. The important factor that quantifies the soil to plant uptake is termed as transfer factor (TF_sp_) defined for the present study as the ratio of alpha activity caused by radionuclides from plants to that of soil. In present work we assumed the following;

1. The alpha activity from the plants is directly proportional to radioactivity contents
2. The range of alpha particle in samples is larger than the thickness of sample so that more than 90 % of alpha produced emit out from the samples.
3. The radioactivity contents in the plants assumed to be small, thus a linear law for transfer factor is applicable.

The transfer factor for soil to plant (TF_sp_) is given by Freundlich equation, which is defined by the non-linear relationship [16, 19]

1 $$ {C}_i={TLF}_{\mathrm{ri}}\cdot {C}_r $$

C_p_ and C_s_ is the alpha track density produced by soil and plants, TF_sp_ the transfer factor and g is factor depends upon the radioactivity contents and hence the alpha activity of soil. For low activity concentration the value of g should be taken equal to one. For the mobility of uranium and their decays products in the plants, another factor called translocation factor from root to other parts of plant (TLF_ri_) factor defined by

2 $$ {\mathrm{C}}_P={TF}_{SP}\cdot {C}_s^g $$

Where i = L,S,F stands for leaf, stem and fruit part of plants. In present work the transfer factor was determined for the alpha track density enhanced by the use of phosphates fertilizers in different age of potato plants grown under control laboratory condition. The potato plant is selected because its pabulum roots are used as major vegetable products all around the world.

## Materials and methods

**Fig. 1:**
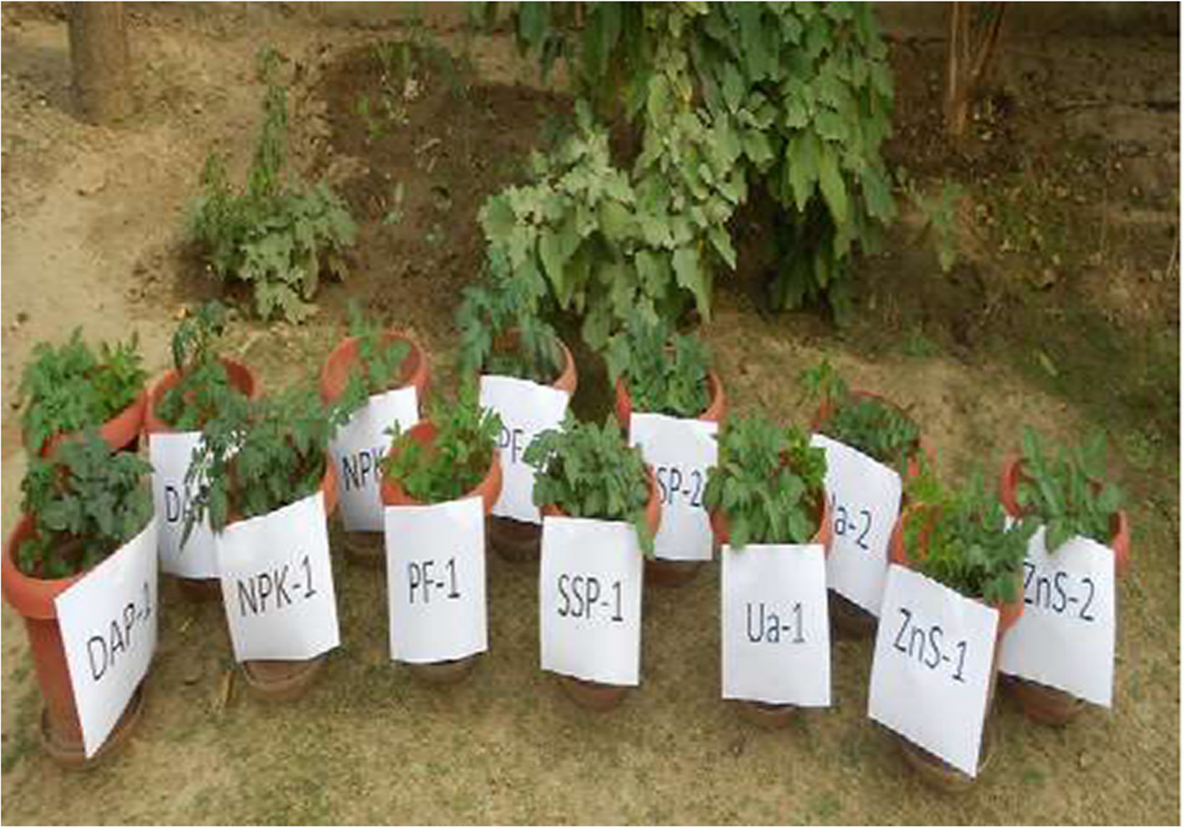
The potato plants at the age of 30 days

The potato (Solanum Tuberosum) plants were grown in earthen pots in control laboratory condition. The soil of the local region was dried at 100^0^ C for 24 h in microwave oven so that most of the moisture of soil was removed and any organic matter present was destroyed. Then the soil left undisturbed to cool for more than a week. A 15 kg soil filtered through 100 micron sieve was filled in 14 different pots of same dimensions having 20 and 50 g of different fertilizer (Diammounium phosphate, Nitrogen phosphate potassium, Phosphate Fertilizer, Single super phosphate, Zinc sulphate and urea) thoroughly mixed in soil. Small seed potatoes were planted a whole, while larger tubers were cut into pieces with 2 to 4 "eyes" on each piece. The seed potatoes gardened at the bottom of a 6 to 10 cm depth of soil from surface and 10 to 15 cm above from lower side of pots. All pots were kept at same open environment condition and similar watering was provided at regular interval of times. Fig. 1 shows the potato plants grown at the age of 30 days. After the 30 and 60 days of plantation, healthy leaves of same size and same portion of the plants were plugged out form each plant, washed thrice with fresh water, so that any contamination on leaves were removed and then dried in normal sunlight for two days. Then leaves were oven dried for 48 hrs at 60 °C, so that most of the moisture was removed. Then each leaf was sandwiched between two SSNTD’s (LR-115, Type-2, Strippable, size 3 × 3 cm^2^), wrapped into aluminum foil sheet and kept into sealed plastic canisters so that it remained isolated from the environment [20]. After the exposure of 60 days the LR-115 detectors were removed and chemically etched in 2.5 N alkaline NaOH solutions at 60^0^ C for 90 mins. The tracks produced by the alpha particles, were counted by spark counter techniques. The etched detectors were first pre sparked at 900 V twice and then tracks were counted at 500 V thrice. The background counts were subtracted from each measurement for alpha track density. The same procedure was repeated for the leaves, stem, roots and fruit part of potato plants at the age of 60 and 105 days. The alpha track density from the fertilized soil and fertilizers was measured by placing SSNTD’s (LR-115) of dimension 2 × 2 cm^2^ over the fertilized soil in pots and fertilizes separately. The measurement of alpha track density measurement from soil using LR-115 was performed three times at age of 30, 60 and 120 days during the growth of plant.

## Result and discussion

Alpha track density from leaves of potato plant at different ages.

Two samples were analyzed for given amount of each fertilizer added to the soil. The alpha track density from the leaves of the potato plants (T cm^−2^d^−1^) measured at the age of 30 days and 60 days are listed in Tables 1 and 2. The alpha track density at 30 days age varied from 0.12 ± 0.01 (0.18 ± 0.04) to 0.43 ± 0.01 (0.81 ± 0.17) for the upper (Lower) part of leaves grown using 20 g of different fertilizers while that for 50 g it varied from 0.13 ± 0.0 (0.24 ± 0.07) to 0.30 ± 0.03 (0.55 ± 0.0) for upper (Lower) part of leaves. The alpha track densities for the leaf part increased with increase in the amount of fertilizers used. This may be because there exist a linear relation between the content of P_2_O_5_ and uranium as both form of uranium [U(SO_4_)_2_] or [UO_2_(SO_4_)] in phosphoric acid (raw materials of fertilizers) is soluble in water [9]. With increase in the amount of fertilizers in pots, increased the phosphorus content in soil [21, 22]. hence, uranium into the plants. Also the alpha track density from the lower parts of the leaves was found to be higher than that of the upper parts, this may be due to high trichrome density in the lower parts of leaves as compared to upper parts [23, 24].

**Table 1:** Alpha Track density per day at the age of 30 days in leaves of potato plants

	Alpha track density per day in leaves (T cm^−2^d^−1^)			
Fertilizer used	Fertilizer used (20 g)		Fertilizers used (50 g)	
	Upper face	Lower face	Upper face	Lower face
Urea	0.12 ± 0.01	0.18 ± 0.04	0.14 ± 0.07	0.24 ± 0.07
Single Super Phosphate	0.23 ± 0.01	0.31 ± 0.03	0.3 ± 0.02	0.45 ± 0.06
NPK	0.2 ± 0.02	0.52 ± 0.07	0.13 ± 0.0	0.55 ± 0.0
Zinc Sulphate	0.43 ± 0.01	0.81 ± 0.17	0.30 ± 0.04	0.34 ± 0.01
Potash fertilizer	0.27 ± 0.05	0.38 ± 0.06	0.28 ± 0.03	0.48 ± 0.04
Diammonium Phosphate	0.41 ± 0.26	0.45 ± 0.23	0.23 ± 0.03	0.47 ± 0.12

**Table 2:** Alpha Track density per day at the age of 60 days in leaves parts of potato plants

	Alpha track density per day in leaves (T cm^−2^d^−1^)			
Fertilizer used	Fertilizer used (20 g)		Fertilizers used (50 g)	
	Upper face	Lower face	Upper face	Lower face
Urea	0.19 ± 0.03	0.29 ± 0.02	0.23 ± 0.06	0.37 ± 0.03
Single Super Phosphate	0.39 ± 0.02	0.43 ± 0.11	0.34 ± 0.02	0.47 ± 0.09
NPK	0.34 ± 0.09	0.35 ± 0.12	0.46 ± 0.15	0.48 ± 0.04
Zinc Sulphate	0.37 ± 0.08	0.62 ± 0.03	0.39 ± 0.06	0.53 ± 0.14
Potash fertilizer	0.38 ± 0.03	0.41 ± 0.11	0.38 ± 0.08	0.88 ± 0.01
Diammonium Phosphate	0.52 ± 0.03	0.29 ± 0.07	0.31 ± 0.03	0.44 ± 0.05

The alpha track density (T cm^−2^d^−1^) at 60 days age varied from 0.19 ± 0.03 (0.29 ± 0.02) to 0.52 ± 0.03 (0.62 ± 0.03) for the upper (lower) part of leaves grown using 20 g of different fertilizers while that for 50 g it varied from 0.23 ± 0.06 (0.37 ± 0.03) to 0.46 ± 0.15 (0.88 ± 0.01) for upper (Lower) part of leaves. The alpha track density in leaves was found to be higher in case of 50 g fertilizers. Also, the alpha track density of the potato plants leaves increased with increase in life of plants. This may be due to longer half life of ^235^U, ^226^Ra and ^210^Pb and available for the transfer from soil to roots and then leaves. The alpha track density from leaves were found to be minimum for the plants grown with urea because the phosphorous content of urea fertilizers was found to be minimum as compare to other fertilizers and maximum for Zinc sulphate (ZnS), diammonium phosphate (DAP) and Nitrogen Phosphorous Potassium (NPK) fertilizers due to their high phosphorous contents measured by Chauhan et al. [24].

The alpha track density from various parts of the potato plants at the age of 105 days of plantation are listed in Table 3. The alpha track density for the root parts (The part of plant underground other than edible part) varied from 0.62 ± 0.16 (0.66 ± 0.19) to 1.52 ± 0.049(1.64 ± 0.06) for the plants grown using 20 g (50 g) of different fertilizers and found to be minimum for urea fertilized plants and maximum for diammonium phosphate and potash fertilized plants. For the stem parts the maximum value of track density was 1.37 ± 0.19 (1.84 ± 0.17), for single phosphate fertilizer using 20 g (50 g) amount.

**Table 3:** Alpha Track density per day at the age of 105 days in root, stem, fruit and leaves of potato plants

Alpha track density per day in root, stem, fruit and leaves (T cm^−2^d^−1^)										
Fertilizers	Root		Stem		Fruit		leaves			
							Upper face	Lower face	Upper face	Lower face
	Fertilizer used (20 g)	Fertilizer used (50 g)	Fertilizer used (20 g)	Fertilizers used (50 g)	Fertilizer used (20 g)	Fertilizer used (50 g)	Fertilizer used (20 g)		Fertilizers used (50 g)	
Urea	SNR	0.66 ± 0.19	SNR	1.01 ± 0.25	0.04 ± 0.01	0.15 ± 0.07	0.74 ± 0.36	0.55 ± 0.02	1.17 ± 0.03	0.51 ± 0.13
Single Super Phosphate	0.77 ± 0.20	1.5 ± 0.12	1.37 ± 0.19	1.84 ± 0.17	0.44 ± 0.19	1.43 ± 0.04	0.48 ± 0.13	0.34 ± 0.03	0.77 ± 0.26	0.96 ± 0.69
NPK	0.98 ± 0.24	1.64 ± 0.06	1.27 ± 0.09	0.51 ± 0.12	0.11 ± 0.03	0.35 ± 0.07	0.44 ± 0.01	0.48 ± 0.03	1.01 ± 0.01	0.96 ± 0.04
Zinc Sulphate	0.91 ± 0.46	1.22 ± 0.05	1.09 ± 0.07	1.47 ± 0.07	0.39 ± 0.04	0.55 ± 0.07	0.63 ± 0.04	0.51 ± 0.13	1.18 ± 0.31	1.04 ± 0.29
Potash fertilizer	0.62 ± 0.16	1.03 ± 0.65	0.89 ± 0.3	1.05 ± 0.09	0.46 ± 0.04	0.72 ± 0.04	0.41 ± 0.01	0.49 ± 0.01	1.34 ± 0.01	0.69 ± 0.04
Di-ammonium Phosphate	1.52 ± 0.04	SNR	1.29 ± 0.37	1.49 ± 0.15	0.2 ± 0.05	0.5 ± 0.1	0.51 ± 0.04	0.40 ± 0.01	0.84 ± 0.13	0.52 ± 0.04
Without fertilizers	SNR	SNR	57 ± 1		0.06 ± 0 .00		0.31 ± 0.14		0.23 ± 0.09	

*SNR* Sample not retrieved

The alpha track density (T cm^−2^d^−1^) in the fruit part (edible parts) of potato plants varied from 0.04 ± 0.01 to 0.46 ± 0.04 for 20 g and 0.15 ± 0.07 to 1.43 ± 0.04 for 50 g amount of fertilizers and again found to be minimum for urea fertilizer. The track density in the leaves of plants showed the similar trends as listed in Tables 1 and 2 and confirmed the accumulations of uranium and their decays products with time. The alpha track density for the plants grown without the use of fertilizers was less than that with fertilizers, which reflects the increase in alpha activity by use of fertilizers.

### Translocation factor

The behaviour of different parts of plants with respect to photo-chemical reaction is not same, therefore the translocation of different nutrient (N, P, K, Fe, etc.) is also not same. The phosphorus is one of the 17 essential nutrients for the growth of leaves, stem as well fruit. The nutrient transfer from soil to roots of the plants is further translocated in the different parts of the plant, which may act as a carrier for uranium and decays products. The translocation factor is the ratio of the activity concentration in the different parts of the plants to that of roots. The calculation of the translocation factor was made to evaluate the ability of potato plants to translocate the radionuclide from root to different part of plants. The leaves of the potato plants are used to feed animals and edible parts (fruit) is used as vegetable all over the world, thus the translocation factor for fruit and leaves part were calculated by using Eq. 1 listed in Table 4.

**Table 4:** Translocation factors* for fruit and leaves of potato plants at age of 105 days

Fertilizers used for plantation	Translocation factor(TLF’s) in			
	Fruit part		Leaf (lower part)	
	20 g fertilizer used	50 g fertilizers used	20 g fertilizer used	50 g fertilizer used
Urea	SNR	0.22	SNR	0.72
Single Super Phosphate	0.57	0.95	0.63	0.64
NPK-1	0.12	0.21	0.48	0.59
Zinc Sulphate	0.42	0.45	0.67	0.85
Potash fertilizer	0.73	0.69	0.66	0.67
Diammonium Phosphate	0.13	-	0.33	-
Average ± SD	0.40 ± 0.26	0.51 ± 0.31	0.55 ± 0.15	0.69 ± 0.14

*Translocation factors in Table 4 were determined by the average value of track density from various parts thus, standard deviation is not included in Table 2

The translocation factor for the fruit (edible Part) varied from 0.13 (for DAP) to 0.73 (for PF) with an average of 0.40 ± 0.26 for the plant grown with 20 g of fertilizers. The translocation factor increased with increase in amount of fertilizers having value 0.51 ± 0.31 for the plant grown with 50 g of fertilizers. The translocation factor for the lower part of leaves grown with 20 g and 50 g fertilizers varied from 0.33 to 0.67 and 0.59 to 0.88 with an average value 0.55 ± 0.15 and 0.69 ± 0.14 respectively. The fruit part of the plants have translocation factor less than that of leaves. This may be because the accumulation of radioactivity in the plant is time dependent. The leaves in the potato plants get developed after 10 days of plantation and accumulation of radionuclide was continuing upto the sampling. On the other hand, the fruit parts of plant get developed after at the age of 40 days, thus, have low alpha activity. The large variation (30-60 %) in the translocation factor for these fertilizers may be due to different chemical behaviour of plants, which may arise due to different pH value of soil. The translocation factors listed in Table 4 represented the combined effect of uranium, radium and their decays products. Rodriguez et al. [19] reported the value of translocation factor 0.00068 ± 0.00009 for uranium and 0.86 ± 0.03 for radium for the sun flower plants. The translocations factor values from present study lies within these two.

### Soil to plant root transfer factor (TF’s)

**Fig. 2:**
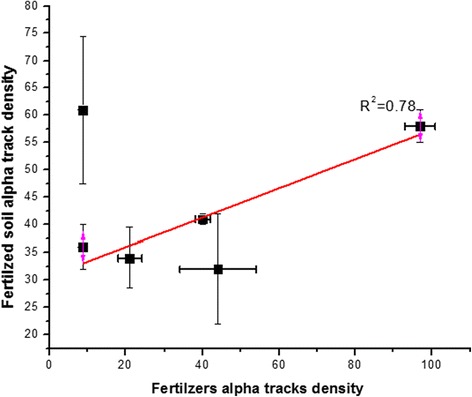
Correlation between the alpha track densities from fertilizers and fertilized soil

The measurement of alpha track density was carried out in duplicate to ascertain the statistical error. The alpha track density per day from the fertilizers and fertilized soil was measured using LR-115 detector and listed in Table 5. The alpha track density from the fertilizers varied from 8.8 ± 0.4 for urea to 97 ± 4 for Diammonium phosphate fertilizers. While track density per day from the fertilized soil samples varied from 49 ± 3 for Zinc sulphate to 98 ± 3 for Diammonium phosphate fertilizers and showed a good correlation (R^2^ = 0.77) with the fertilizer alpha track density (Fig. 2). The transfer factors defined by Eq. 2 were calculated in order to measure the ratio of radionuclides transfer from soil to various parts of plants (Table 6). The measurement of the transfer factor was carried out from the average value of track density in Tables 1, 2, 3 and 5, therefore, the standard deviation and errors are not reported in Table 6.

**Table 5:** Alpha track density from the fertilizers and fertilize soil

	Alpha track density from the		
Fertilizers used	Fertilizers	Fertilized soil (20 g)	Fertilized soil (50 g)
Diammonium Phosphate	97 ± 4	57.5 ± 3	98 ± 3
NPK	44 ± 10	32 ± 10	84 ± 1
Potash fertilizer	40 ± 2	41 ± 1	65 ± 7.5
Single super phosphate	21 ± 3	33.5 ± 5.5	34.5 ± 6.5
Urea	8.8 ± 0.4	60.5 ± 13.5	59.5 ± 6.5
Zinc Sulphate	8.8 ± 1.1	35.5 ± 4	49 ± 3

**Table 6:** Transfer factor from different parts of potato plants at age of 105 days

Fertilizers	Root	Stem	Fruit	Lower side of leaves
Diammonium Phosphate	1.6 × 10^−2^	1.3× 10^−2^	2.1× 10^−3^	5.4 × 10^−3^
NPK	2.2× 10^−2^	2.9 × 10^−2^	2.5× 10^−3^	2.2× 10^−2^
Potash fertilizer	1.5× 10^−2^	2.6 × 10^−2^	1.2 × 10^−2^	1.7× 10^−2^
Single super phosphate	3.7× 10^−2^	6.5 × 10^−2^	2.1 × 10^−2^	4.6 × 10^−2^
Urea	SNR	SNR	4.5× 10^−2^	5.8 × 10^−2^
Zinc Sulphate	1.0 × 10^−1^	1.2 × 10^−1^	4.4× 10^−2^	1.9× 10^−2^

*SNR* Sample not retrieved

**Fig. 3:**
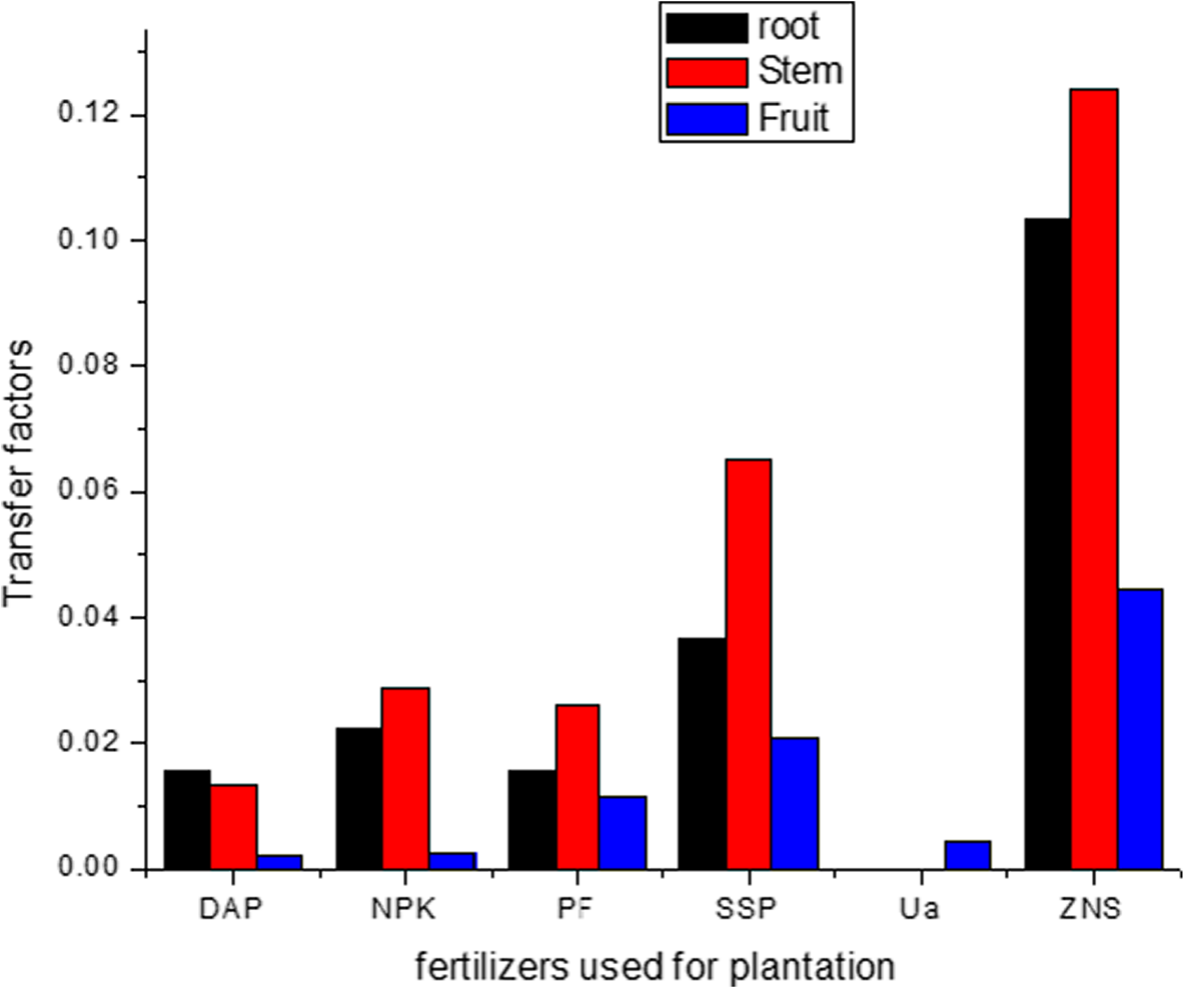
Transfer factor from different parts of potato plant at the age of 105 days

**Fig. 4:**
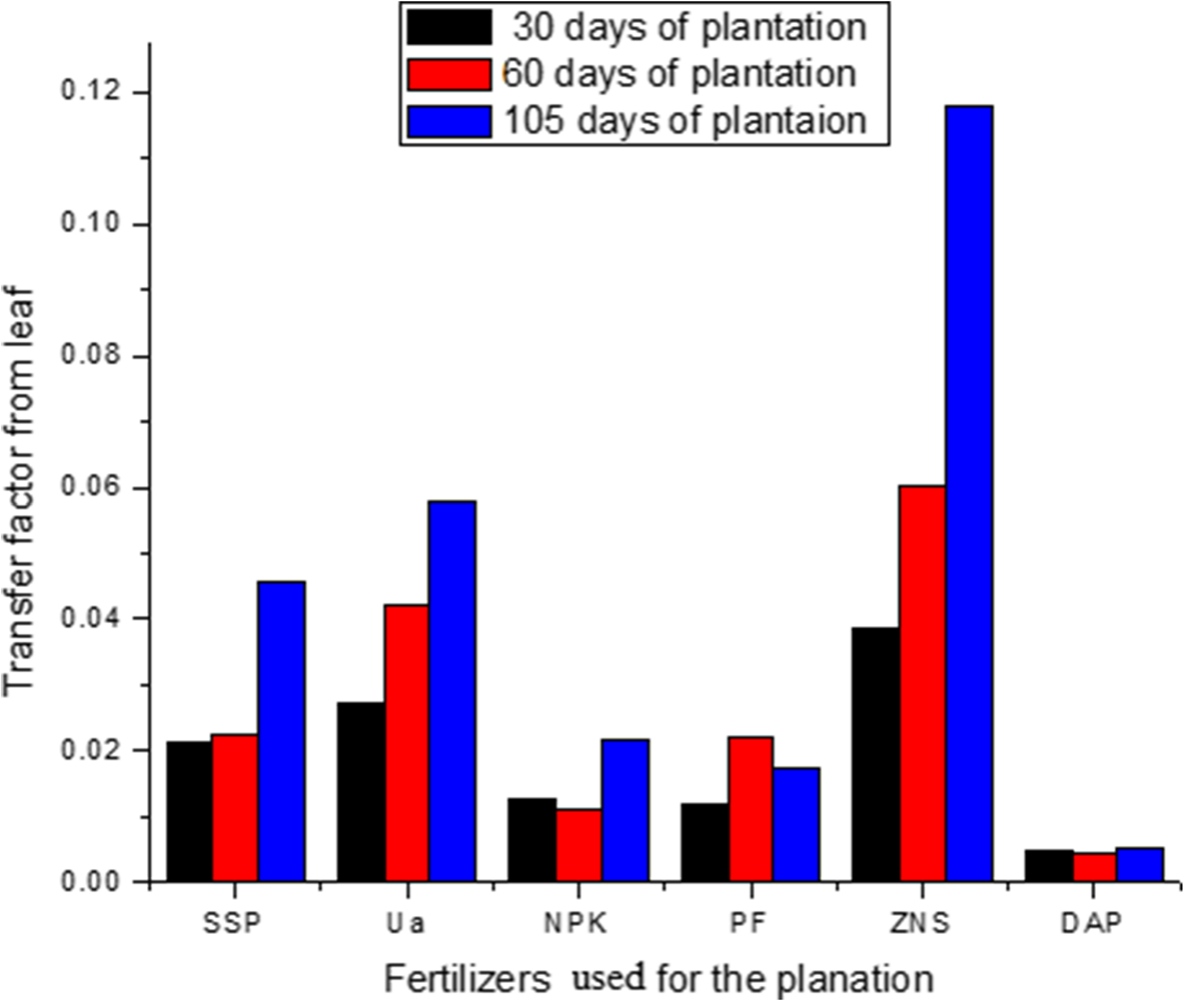
TF’s value from lower part of leaf at different age of potato plant

The TF’s value for potato plants varied from 1.5 × 10^−2^ to 1.0 × 10^−1^ for root, from 1.3 × 10^−2^ to 1.2 × 10^−1^ for stem, from 2.1 × 10^−3^ to 4.5 × 10^−2^ for fruit and from 5.4 × 10^−3^ to 5.8 × 10^−2^ for lower part of leaves at the age of the 105 days of the potato plants using different fertilizers. A comparison of the TF’s value from various parts of plant grown using different fertilizers is shown in Fig. 3. The TF’s value from the lower part of the leaves of potato plant at different age of the plants were also compared and shown in Fig. 4.

The alpha activity from the fruit (edible) parts of the potato plants was smaller than the roots in spite of their underground existence. The root’s hair under the ground provide the large surface to volume ratio for the uptake of element and water and the edible parts life under the ground was smaller to accumulate the radioactivity content than that of the root. Due to same reason the alpha activity from the stem and leaves parts are also higher than fruit parts. The increase in the alpha activity in the leaves of potato plants evidence the time accumulation of radioactivity. Different plant reaction to the uptake of elements is different depending upon the condition of growth, chemistry and climate regime. The uranium which is non essential element usually accumulates in the roots of the plants due to *casparian strip* [25], thus evidence the occurrence of high alpha activity in the roots in present study. While due to the different chemical behaviour of radium as that of uranium and long time necessary for successful *phytoextraction*, it migrates in the different parts of plant and causes high alpha activity in stem and leaves part. Soudek et al. [26] and Masri et al. [27] reported the TF’s value from 0.9 to 7.8 for ^40^ K, 0.3 × 10^−2^ to 1.2 × 10^−1^ for ^238^U and 2.8 × 10^−2^ to 9.1× 10^−2^ for ^210^Po and ^210^Pb for stem, leaves and fruit of different plants species. The TF’s value reported for leaves parts was higher than that of fruit parts for olives, apple and grapes. Soudek et al. [26] reported the TF’s value from 1.3 × 10^−2^ to 1.6 × 10^−1^ for 28 different species grown in green house and confirmed the nearly linear behaviours of element from soil to plant uptake. The alpha activity in the different parts of plant considered in the present study was assumed to be caused by the radionuclide uranium and radium along with their decay products thus the TF’s value lie in the range reported in previous studies. The measurement of TF’s value for five boreal forest species was carried out by Tuovinen et al. [16] and reported that the assumption for the linear increase of element (Constant TF’s value) did not hold. The element concentration ratio remains symmetrical and decreased with increase in the element concentration in soil. The applicability of linear and non linear assumptions is not unique for specific plants and elements. Sheppard [28] considered linearity assumption valid for range of elements concentrations in the soil is 5 orders of magnitude. Sheppard and Sheppard [29] studied the transfer factor for natural uranium, and concluded that the linearity assumption is valid only for concentrations higher than 1.8 × 10^6^ Bq/kg. Blanco Rodrıguez et al. [18] reported that the linearity assumption for ^238^U, ^230^Th and ^226^Ra is valid for two orders of magnitude of range concentrations of elements. In present study the alpha activity from the potato plants varied two orders of magnitude, thus linearity assumption for transfer factor was applied. The result of TF’s value for potato plants agreed with the range published in literature for different plant species.

A linear and non linear assumption for the soil to root uptake of stable and radioactive elements is not only the function of elements concentration in soil but the other factors like the physiology of plants with reference to particular elements, condition of growth, and metabolism mechanism altered by the usage of fertilizers, soil property and uptake of water also affect the TF’s value thus, includes a lot of uncertainty [2]. This means a single model for transfer factor cannot be applied for group of plants species, even belonging to same family. In the absence of broadly applicable and low-variance models for plant uptake, site-specific models should be derived for definitive risk assessments intended to support remediation decisions which would be applicable to the soil type(s), variety of plant taxa, and variety of chemical forms at a contaminated site [30].

## Conclusions

Radioactivity is always present in the soil and inspite of the low concentration of these elements; they significantly increase the radioactivity in the ecosystem. Therefore the present study has been regarded as of vital importance. The alpha activity from various parts of the potato plants was found to be higher in case of phosphate fertilizers used compared with others. This may have been caused by the presence of uranium and radium in the phosphate rocks; raw materials used for the fertilizers production. The translocation factors from root to leaves and fruit (edible parts) were also calculated. The translocation factors for the fruit (edible parts) of the potato plants grown with different fertilizers were found to be lower than root hair. This may be explained on the basis of small residency time of the fruit as compared to root hair, remaining underground along with the plants.
